# Mapping of Functional Subdomains in the *at*ALKBH9B m^6^A-Demethylase Required for Its Binding to the Viral RNA and to the Coat Protein of Alfalfa Mosaic Virus

**DOI:** 10.3389/fpls.2021.701683

**Published:** 2021-07-05

**Authors:** Luis Alvarado-Marchena, Joan Marquez-Molins, Mireya Martinez-Perez, Frederic Aparicio, Vicente Pallás

**Affiliations:** Instituto de Biología Molecular y Celular de Plantas, Consejo Superior de Investigaciones Científicas, Universidat Politècnica de Valencia, Valencia, Spain

**Keywords:** *N*cpsdummy6-methyladenosine, RNA covalent modifications, RNA-binding proteins, plant viruses, alfamovirus, RNA demethylases, epitranscriptomics, ALKBH

## Abstract

*N*^6^-methyladenosine (m^6^A) modification is a dynamically regulated RNA modification that impacts many cellular processes and pathways. This epitranscriptomic methylation relies on the participation of RNA methyltransferases (referred to as “writers”) and demethylases (referred to as “erasers”), respectively. We previously demonstrated that the Arabidopsis thaliana protein *at*ALKBH9B showed m^6^A-demethylase activity and interacted with the coat protein (CP) of alfalfa mosaic virus (AMV), causing a profound impact on the viral infection cycle. To dissect the functional activity of *at*ALKBH9B in AMV infection, we performed a protein-mapping analysis to identify the putative domains required for regulating this process. In this context, the mutational analysis of the protein revealed that the residues between 427 and 467 positions are critical for *in vitro* binding to the AMV RNA. The *at*ALKBH9B amino acid sequence showed intrinsically disordered regions (IDRs) located at the N-terminal part delimiting the internal AlkB-like domain and at the C-terminal part. We identified an RNA binding domain containing an RGxxxRGG motif that overlaps with the C-terminal IDR. Moreover, bimolecular fluorescent experiments allowed us to determine that residues located between 387 and 427 are critical for the interaction with the AMV CP, which should be critical for modulating the viral infection process. Finally, we observed that *at*ALKBH9B deletions of either N-terminal 20 residues or the C-terminal’s last 40 amino acids impede their accumulation in siRNA bodies. The involvement of the regions responsible for RNA and viral CP binding and those required for its localization in stress granules in the viral cycle is discussed.

## Introduction

The addition of a methyl group to the N^6^ position of adenosine (m^6^A) is the most abundant internal modification in eukaryote mRNAs (Boccaletto et al., 2018; Covelo-Molares et al., 2018; Arribas-Hernández and Brodersen, 2020; Zhou et al., 2020). It regulates many steps of RNA metabolism, including splicing (Zhao et al., 2014), stability (Wang et al., 2014), translation (Meyer et al., 2015), nuclear-export (Zheng et al., 2013), RNA structures (Bayoumi et al., 2020), and protein/RNA interactions (Liu et al., 2015). Also, it modulates the epigenetic effects of some non-coding RNAs (ncRNA) (Meyer and Jaffrey, 2017). Since the 1970s, m^6^A modification has been known to tag not only cellular RNAs but also RNAs of multiple viruses (Wei and Moss, 1975; Furuichi et al., 1976; Krug et al., 1976; Dimock and Stoltzfus, 1977), although its functional relevance has remained elusive mainly due to the lack of efficient methods of m6A detection and subsequent analysis. Recent studies have demonstrated the crucial roles of m^6^A in the virus–host interactions; however, most of these studies have focused on animal viruses (Dang et al., 2019; Williams et al., 2019; Arribas-Hernández and Brodersen, 2020), whereas it remains very limited in plant viruses (Martínez-Pérez et al., 2017; Li et al., 2018; Arribas-Hernández and Brodersen, 2020).

In mammals, m^6^A methylation is catalyzed co-transcriptionally by a multicomponent m^6^A methyltransferase complex (MTC, also known as “writer”) (Liu et al., 2014; Ping et al., 2014). The core component of MTC is a ∼200 kDa heterodimer comprised of METTL3 and METTL14 (Huang et al., 2020). Other regulatory subunits of MTC have also been identified, including WTAP and its cofactors KIAA1429 (VIRMA), ZC3H13, and RBM15/RBM15B, which play roles in anchoring MTC to nuclear speckles and U-rich regions adjacent to m^6^A sites in mRNAs. Two main RNA demethylases, “erasers,” belonging to Fe(II)/2-oxoglutarate (2OG) dioxygenase superfamily, named AlkB homology 5 (ALKBH5) and FTO, have been described to remove m^6^A marks (Aik et al., 2014; Xu et al., 2014). The third component of the m^6^A modification machinery consists of “reader” proteins that recognize this modification and modulate the activity and half-life of diverse RNAs. Thus, several YTH domain family members, YTHDF1, YTHDF2, YTHDF3, YTHDC1, and YTHDC2, mediate many of the phenotypic effects of this epitranscriptomic modification (Luo and Tong, 2014; Xiao et al., 2016).

By homology with mammals, functional orthologous genes of the m^6^A machinery have been discovered in *Arabidopsis* (Arribas-Hernández and Brodersen, 2020). MTA, MTB, FIP37, VIRILIZER, orthologs of METTL3, METTL14, WTAP, and KIAA1429 (human protein), respectively, have been identified as member proteins of the m^6^A *writer* complex (Zhong et al., 2008; Bodi et al., 2012; Shen et al., 2016; Růžička et al., 2017). In the *Arabidopsis* genome, 14 “readers” of the YTH family have been identified (ECT1-11; Evolutionarily Conserved C-Terminal Region 1–11, At4g11970 and the Cleavage and Polyadenylation Specificity Factor 30). The *Arabidopsis* genome encodes 14 homologs of Alk B family of “eraser” proteins (*at*ALKBH1A-D, *at*ALKBH2, *at*ALKBH6, *at*ALKBH8A-B, *at*ALKBH9A-C, and *at*ALKBH10A-B) (Mielecki et al., 2012; Kawai et al., 2014), of which, *at*ALKBH9B and *at*ALKBH10B, have been shown to present m^6^A demethylase activity *in vitro* and m^6^A-related functions *in vivo* (Duan et al., 2017; Martínez-Pérez et al., 2017). Recently the potential *eraser at*ALKBH6 has been shown to play important roles in seed germination, seedling growth, and survival of *Arabidopsis* under abiotic stresses (Huong et al., 2020). Interestingly, *at*ALKBH9B is the only m^6^A demethylase that is located exclusively in the cytoplasm (Mielecki et al., 2012), forming granules that colocalize with SGS3 (a component of siRNA bodies) and in some cases, associates with DCP1 (P bodies) (Ingelfinger et al., 2002; Martínez de Alba et al., 2015; Martínez-Pérez et al., 2017).

*Arabidopsis* genome has been described to encode more than 200 putative RNA-binding proteins (RBP) (Lorković and Barta, 2002; Abbasi et al., 2011; Ambrosone et al., 2012). RBPs are key factors in post-transcriptional gene regulation, protein synthesis, viral replication, cellular defense, and developmental regulation (Terribilini et al., 2006; Glisovic et al., 2008; Pallas and Gómez, 2013; Prall et al., 2019). RBPs are often modular and contain one or more conserved RNA-binding domains (RBD) (Lee et al., 2012; Re et al., 2014). RNA recognition motifs (RRM) and the K homology (KH) domain are the most abundant structural motifs in eukaryotes (Lorković and Barta, 2002; Chen and Varani, 2013). Other RBDs include the glycine-rich motif (GRM), the double-stranded RNA binding domain (dsRBD), DEAD- Box-, PUF, SAM-, ZnF-domains (Lunde et al., 2007; Re et al., 2014; Lee and Kang, 2016), and the RGG/RG motif (Thandapani et al., 2013). However, the majority of residues predicted to be in the protein-RNA interface are not part of a characterized RBDs (Terribilini et al., 2006). In many cases, intrinsically disordered regions (IDRs) have been identified in proteins that do not display characterized RNA-binding sites (Calabretta and Richard, 2015; Varadi et al., 2015). Among other roles, IDRs participate in protein-protein and protein-RNA interactions and are enriched in “disorder-promoting amino acids” such as G, P, or R (Gsponer and Madan Babu, 2009). In this context, IDRs can encompass diverse functional motives such as RNA binding motifs or low-complexity (LC) domains (Castello et al., 2012). These proteins often go through binding induced-folding. Thus, as a consequence of their structural felxibility, the RNA-protein interactions can experiment conformational changes in the protein structure, the RNA or both (Frankel, 1999). Additionally, proteins containing IDRs promote liquid-liquid phase separation in the assembly and degradation of RNA granules such as stress granules or P-bodies (Spector, 2006; Lin et al., 2015). Similar to what was observed in mammals, some *Arabidopsis* m^6^A *readers*, such as ECT2, ECT3, and ECT4 present IDRs and can form cytoplasmic granules (Scutenaire et al., 2018; Arribas-Hernández et al., 2020). Moreover, ECT2 was found to undergo a gel-like phase transition *in vitro* (Arribas-Hernández et al., 2018).

Alfalfa mosaic virus (AMV) belongs to the *Bromoviridae* family and, like the rest of the members of this family, its genome consists of three single-stranded RNAs of plus polarity (Bujarski et al., 2019). RNA1 and RNA2 encode the replicase subunits (P1 and P2), whereas RNA3 encodes the movement protein (MP) and serves as a template for the synthesis of subgenomic RNA4 (sgRNA4), which encodes the coat protein (CP) (Bol, 2005; Pallas et al., 2013). We previously demonstrated that *at*ALKBH9B is a key factor in AMV infection since the suppression of the *at*ALKBH9B m^6^A demethylation activity reduces viral accumulation. Moreover, it was shown that the CP of this virus interacts with *at*ALKBH9B, pointing to a direct subversion of an endogenous regulatory pathway by the virus (Martínez-Pérez et al., 2017). Due to the functional relevance of *at*ALKBH9B, the first m^6^A demethylase described in plants, we carried out a functional mapping of the protein to identify putative domains implicated in the diverse interactions of this m^6^A-demethylase with both the viral RNA and the coat protein.

## Results

### *In vitro* Mapping of the RNA-Binding Domain of *at*ALKBH9B

**FIGURE 1:**
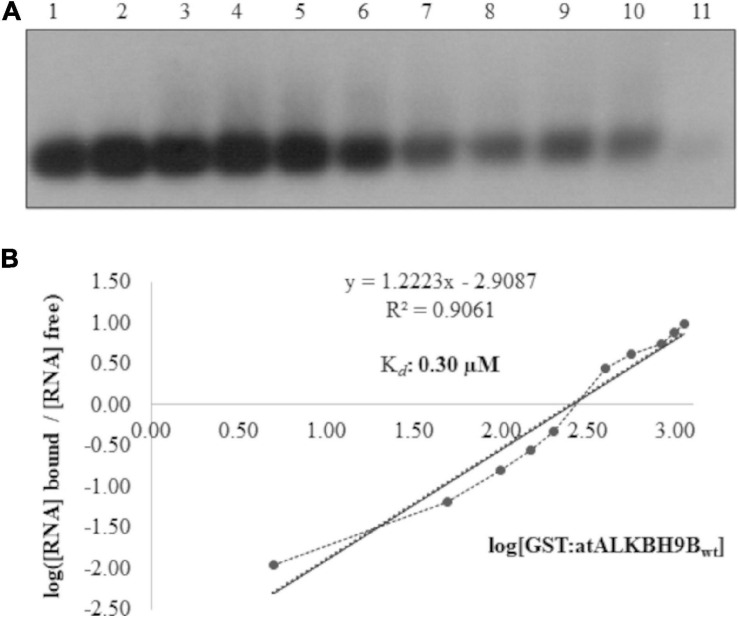
Analysis of RNA-protein complexes formed between purified GST:*at*ALKBH9B_wt_ protein and the 3′UTR transcript of AMV-RNA3/4. **(A)** EMSA after incubation of 5 ng of 3′UTR transcript with no protein (lane 1) or with 5, 50, 100, 150, 200, 400, 560, 840, 980, and 1120 ng of GST:*at*ALKBH9B_wt_ (lanes 2–11) corresponding to 0.01, 0.06, 0.12, 0.18, 0.24, 0.48, 0.67, 1.00, 1.17, and 1.22 μM, respectively. **(B)** Hill transformation. The thin line is the best fit determined by least-squares analysis, with the corresponding *r* coefficient and equation given in the insert. The slope of the best-fit equation determines a Hill coefficient of 1.35, indicating no or mild cooperativity, and the *y*-intercepted point gives the value of the K*_d_*, which was 0.30 μM.

In a previous study, we demonstrated that *at*ALKBH9B interacts with the viral RNA, although the kinetic parameters of this interaction, as well as the identification of the RBD, were not analyzed (Martínez-Pérez et al., 2017). To estimate the capacity of the wild-type *at*ALKBH9B to bind the viral RNA, we first conducted Electrophoretic Mobility Shift Assays (EMSA) by incubating a constant amount (5 ng) of an RNA transcript, corresponding to the 3′ untranslated region of AMV-RNA3 (3′UTR-RNA3), with increasing concentrations of the glutathione S-transferase fusion protein (GST:*at*ALKBH9B_wt_) (Figure 1). We chose this part of the RNA3/4 molecule because it is the same as the one that specifically binds CP, allowing us to directly compare it with this specific interaction. The decrease in the chemiluminescent signal intensity corresponding to free RNA was evident at quantities exceeding 400 ng of GST:*at*ALBKH9B_wt_ (lane 7, Figure 1A), suggesting the formation of a protein–RNA complex. At this point, it is important to note that the non-radioactive EMSA described here (see section “Material and Methods”) requires the transfer to a nylon membrane step to detect both free RNA and the corresponding ribonucleoprotein (RNP) complex. When a large amount of molecules binds to the RNA, as in a non-sequence specific interaction, the transfer of the RNP complex to the nylon membrane and its posterior detection is difficult or even impossible (Marcos et al., 1999; Herranz and Pallás, 2004; Salavert et al., 2020). In any case, the disappearance of the free RNA band is evidence of complex formation (Carey, 1991) and was therefore quantified by film densitometry to calculate the apparent constant dissociation (Kd) of the RNA– GST:*at*ALBKH9Bwt interaction from the linear regression of the mean values from at least three technical replicates (Marcos et al., 1999). The K*_d_* value of GST:*at*ALKBH9B_wt_ was estimated to be 0.30 μM (Figure 1B).

**FIGURE 2:**
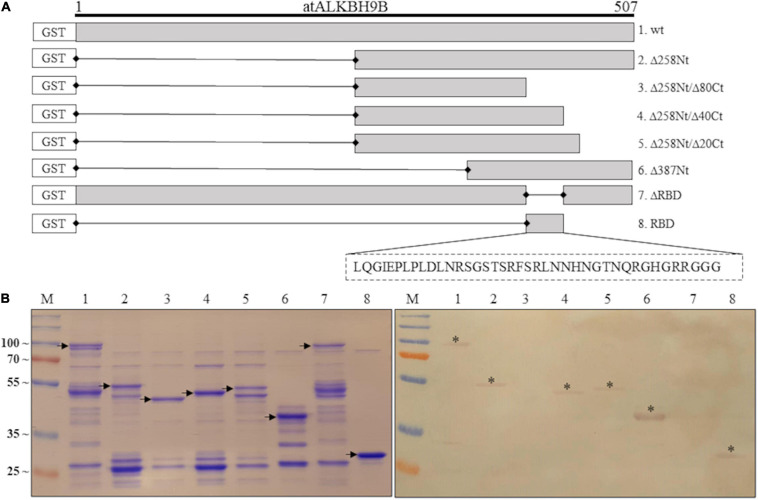
Analysis of the RNA-binding activity of purified GST:*at*ALKBH9B proteins by Northwestern blot assay. **(A)** GST fusion proteins containing Internal or C-terminal deletions of *at*ALKBH9B were produced in a prokaryotic system. **(B)** Duplicated membranes with GST:*at*ALKBH9B_wt_ (lane 1) or its mutant variants (lanes 2–8), as depicted in **(A)**, were resolved by 12% SDS–PAGE and, after transferring to nitrocellulose, incubated with 5 μg of AMV-sgRNA 4 labeled with digoxigenin. The left panel shows a Coomassie blue-stained gel, and the right panel shows results from Northwestern blot analysis. Positions of full-length GST:*at*ALKBH9B proteins are indicated by arrows. Asterisks indicate GST:*at*ALKBH9B fusion proteins which interact with the AMV-sgRNA4. The positions of molecular mass markers (in kDa) are shown on the left side.

In order to determine the region of *at*ALKBH9B_wt_ with RNA-binding activity, we first designed GST:*at*ALKBH9B deletion mutants lacking 160 amino acids at either the N- or C-terminal part (Δ160Nt and Δ160Ct, respectively), or 187 amino acids in the internal region of the protein (Δ187 Int) (Supplementary Figure 1A). Northwestern blot assays using AMV-sgRNA4 labeled with digoxigenin showed a complete loss of RNA-binding capacity of GST:*at*ALKBH9B_Δ__187Int_ and GST:*at*ALKBH9B_Δ__160Ct_ proteins (Supplementary Figure 1B). Thus, we designed new GST:*at*ALKBH9B deletion mutants affecting the internal and/or C-terminal regions: GST:Δ258Nt; GST:Δ258Nt/Δ80Ct; GST:Δ258Nt/Δ40Ct; GST:Δ258Nt/Δ20Ct and GST:Δ387Nt (Figure 2A). Northwestern blot assays showed that only mutant Δ258Nt/Δ80Ct does not bind RNA, indicating that residues between positions 427 and 467 are critical for RNA-protein interaction (Figure 2B). To confirm this observation, we evaluated the RNA-binding activity of a mutant lacking residues from 427 and 467 (GST:ΔRBD) and one comprising only the *at*ALKBH9B 427–467 residues (GST:RBD) (Figure 2A). As shown in Figure 2B, only GST:RBD retained the RNA-binding capacity.

**FIGURE 3:**
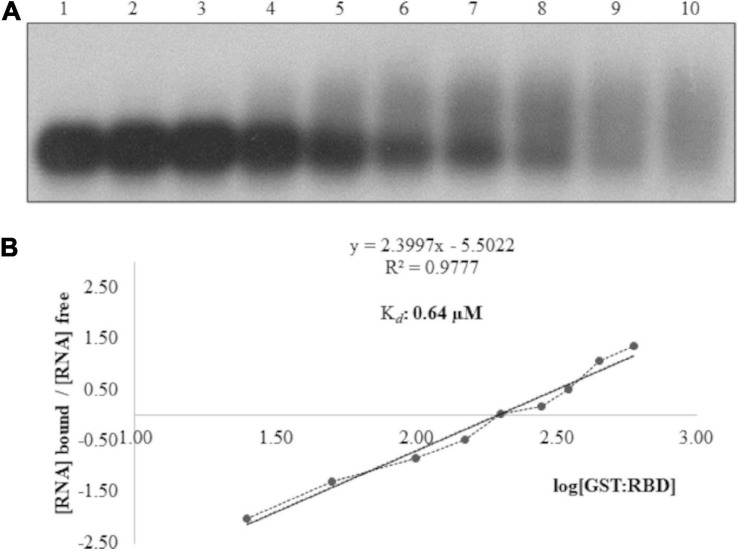
Analysis of RNA–protein complexes formed between purified GST:RBD of *at*ALKBH9B protein and the 3’UTR transcript of AMV-RNA3/4. **(A)** EMSA after incubation of 5 ng of 3’UTR transcript with no protein (lane 1) or with 25, 50, 100, 150, 200, 280, 350, 450 and 600 ng of GST:RBD of 9B (lanes 2–10) corresponding to 0.08, 0.16, 0.33, 0.49, 0.65, 0.91, 1.14, 1.47, and 1.96 μM, respectively. **(B)** Hill transformation. The thin line is the best fit determined by least-squares analysis, with the corresponding *r* coefficient and equation given in the insert. The slope of the best-fit equation determines a Hill coefficient of 2.4, and the *y* intercepted point gives the value of the apparent constant dissociation (K*_d_*), which was 0.64 μM.

To validate the northwestern blot assays, EMSAs were performed with GST:*at*ALKBH9B_wt_, GST:Δ160Nt, GST:Δ258Nt; GST:Δ258Nt/Δ80Ct; GST:Δ258Nt/Δ40Ct and GST:Δ258Nt/Δ20Ct by incubating the viral RNA with different protein concentrations. As expected, EMSA showed a decrease in chemiluminescent signal intensity corresponding to the free viral RNA in mutants containing the RBD (Supplementary Figures 2A,B), whereas those lacking the predicted RBD did not reveal RNA binding (GST:Δ258Nt/Δ80Ct). Additionally, we studied the affinity and specificity of the *at*ALKBH9B RBD-RNA interaction by analyzing the shift in electrophoretic mobility of the 3’UTR-RNA3 (AMV) in the presence of GST:RBD fusion protein. As shown in Figure 3, increasing concentrations of GST:RBD diminished the amount of free RNA that becomes undetectable at 450 ng of the protein. A Hill transformation was used to analyze our data (Figure 3B). The K*_d_* value of the RBD fused to GST was 0.64 μM, indicating slightly less RNA-binding than the full-length protein (GST:*at*ALKBH9B_wt_). From the linear regression adjustment, a Hill coefficient c of 2.4 was obtained; this value above 1 (*c* = 1 indicates no cooperativity) would be taken as an indication of positive cooperativity.

**FIGURE 4:**
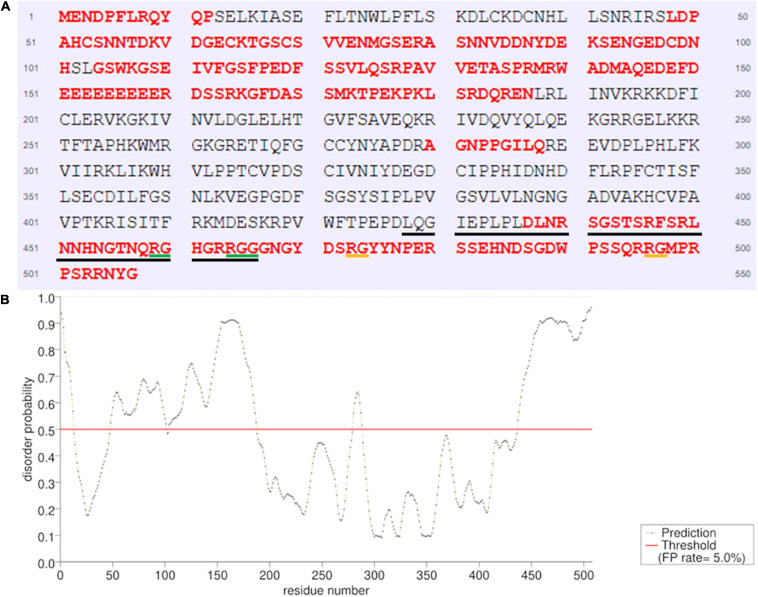
Prediction of natively disordered regions of *at*ALKBH9B using PrDOS. **(A)** *at*ALKBH9B amino acid sequence with disordered residues in red. The RBD located between positions 427–467 is underlined (black line). RGxxxRGG motif is underlined in green and the two extras RGs are in orange. **(B)** Disorder profile plot. Prediction false positive rate: 5%.

Subsequently, we used PSI-BLAST (Altschul et al., 1997) to compare *at*ALKBH9B RBD with other RNA binding proteins (RBP) from the databank, but the alignment of sequences did not reveal any significant similarities. Nonetheless, a high percentage of IDRs has been reported in viral, prokaryotic, and eukaryotic RBPs (Varadi et al., 2015). Therefore, we evaluated whether *at*ALKBH9B presented these IDRs, using PrDOS^1^ (Ishida and Kinoshita, 2007). The results showed that 45.4% of all amino acids of *at*ALKBH9B form IDRs through both N- and C-terminal regions, including most RBD (aa 427–467) (Figure 4). Remarkably, this RBD is enriched in G and R residues (20% and 15%, respectively), which are also common in the aforementioned IDRs. It is worth noting the presence of a RGxxxRGG motif and two extras RG residues that have been described in several RBPs showing characteristic disorder regions (Thandapani et al., 2013).

Taken together, these results indicate that *at*ALKBH9B efficiently cooperatively binds viral RNA through an RBD that has the characteristics of the IDRs present in other viral and eukaryotic RBPs.

### *In vivo* Mapping of *at*ALKBH9B Binding Domain to AMV CP

**FIGURE 5:**
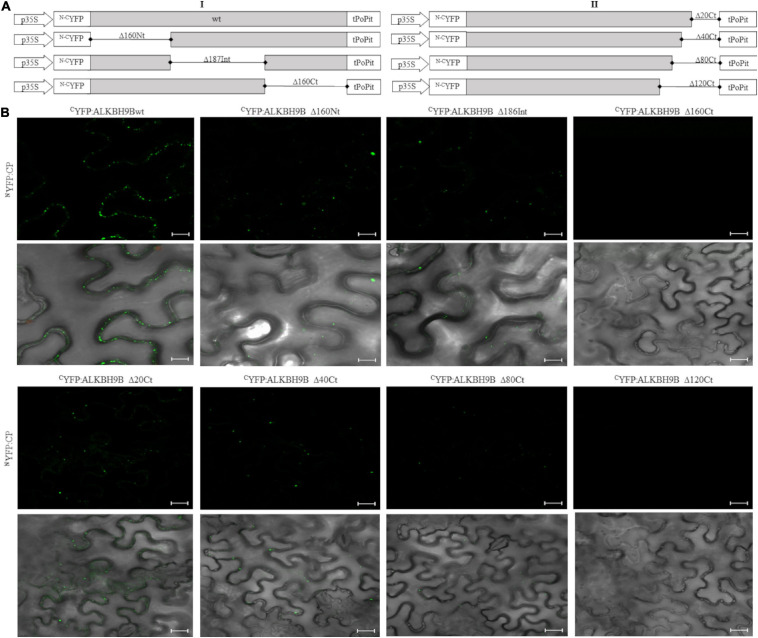
*In vivo* BiCF visualization of the interaction between ^*N–C*^YFP:*at*ALKBH9B fusion proteins and ^*N–C*^YFP:CP of AMV in *N. benthamiana*. **(A)** Schematic representation of the ^*N–C*^YFP:*at*ALKBH9B fusion proteins. **(B)** BiFC images of epidermal cells co-infiltrated with ^*C*^YFP:*at*ALKBH9B proteins combined with the ^*N*^YFP:CP, previously described in Aparicio et al. (2006). Scale bar = 20 μm.

We previously showed that the demethylase activity of *at*ALKBH9B affected the infectivity of AMV but not of cucumber mosaic virus (CMV), correlating with the ability of *at*ALKBH9B to interact (or not) with their coat proteins (Martínez-Pérez et al., 2017). Since this interaction is, thus, critical for the proviral function of *at*ALKBH9B, we decided to delineate the domain of *at*ALKBH9B involved in the host-protein/viral-protein interaction using the Bimolecular Fluorescent Complementation (BiFC). For this purpose, we first designed three mutants corresponding to the N-terminal, central and C-terminal regions, fused to the N- and C-terminal parts of the YFP (Figure 5A, left panel [I]). These constructs were co-infiltrated with the corresponding ^*N–C*^YFP:CP proteins in *Nicotiana benthamiana* leaves, and fluorescence was examined by confocal laser scanning microscopy (CLSM) after 48h. Leaves co-infiltrated with ^*C*^YFP-9B_wt_ plus ^*N*^YFP-CP, ^*C*^YFP-9B_Δ__160Nt_ plus ^*N*^YFP-CP, and ^*C*^YFP:9B_Δ__187Int_ plus ^*N*^YFP-CP rendered a strong YFP fluorescence signal in the cells, whereas no reconstituted YFP fluorescence was detected in leaves co-infiltrated with the pair ^*C*^YFP-9B_Δ__160Ct_ plus ^*N*^YFP:CP (Figure 5B and Supplementary Figure 3, upper panels).

To further delimit the domain involved in *at*ALKBH9B/AMV-CP interaction, we designed new ^*N–C*^YFP:fusion mutants by deleting residues located at the C-terminal region of *at*ALKBH9B (Figure 5A, right panel [II]). Leaves co-infiltrated with ^*C*^YFP-*at*ALKBH9B_Δ__20Ct_ plus ^*N*^YFP:CP, ^*C*^YFP:*at*ALKBH9B_Δ__40Ct_ plus ^*N*^YFP:CP and ^*C*^YFP:*at*ALKBH9B_Δ__80Ct_ plus ^*N*^YFP:CP produced a reconstituted YFP fluorescence signal in the cells, whereas no YFP fluorescence was detected in leaves co-infiltrated with the pair ^*C*^YFP:*at*ALKBH9B_Δ__120Ct_ plus ^*N*^YFP:CP (Figure 5B and Supplementary Figure 3, lower panels). Finally, western blot assays using anti ^*c*^YFP and ^*N*^YFP antibodies confirmed that all *at*ALKBH9B mutated versions fused to the ^*c*^YFP and the ^*N*^YFP:CP accumulated at detectable levels in the co-infiltrated tissues (Supplementary Figure 4). Overall, our results suggest that amino acids located between positions 387 and 427 of *at*ALKBH9B are critical for the interaction with the AMV-CP.

### The Subcellular Localization of *at*ALKBH9B Depends on the Correct Folding of the Protein

*at*ALKBH9B is the only protein of the 14 homologs of *E. coli* AlkB that is exclusively localized in the cytoplasm (Mielecki et al., 2012). Our previous studies determined that *at*ALKBH9B can specifically colocalize with SGS3 protein (a component of siRNA bodies), forming biomolecular-condensates associated with stress granules (Martínez-Pérez et al., 2017).

**FIGURE 6:**
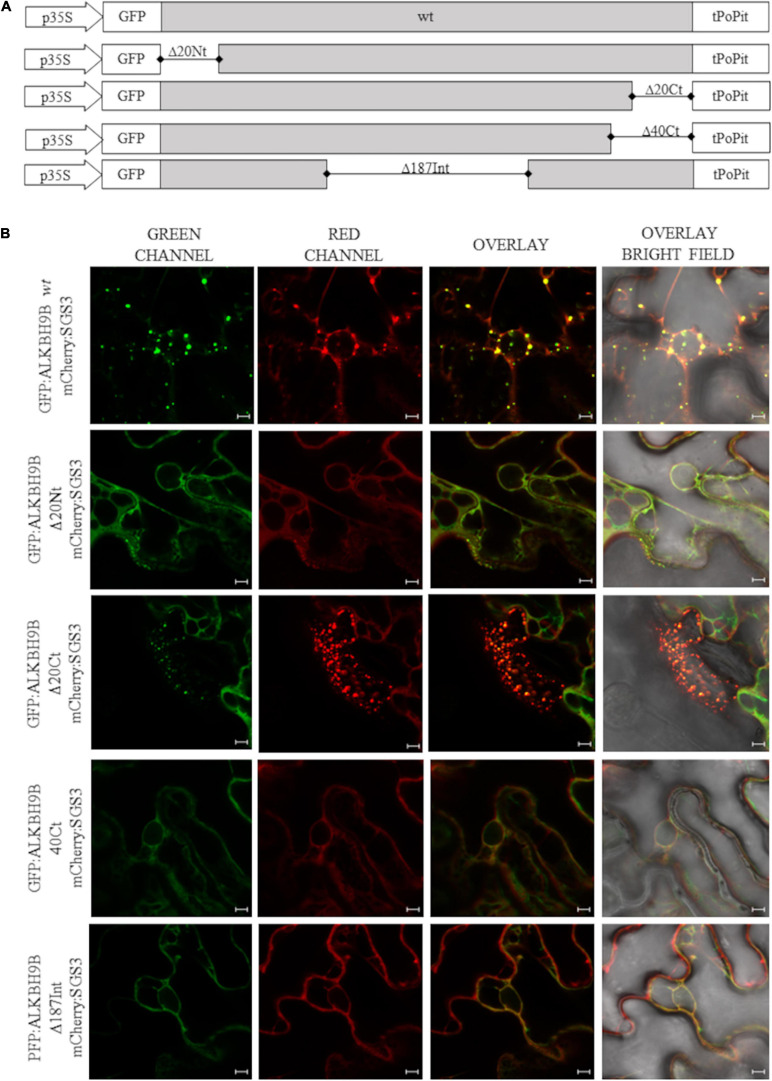
Subcellular localization of *at*ALKBH9B proteins fused with GFP in infiltrated leaves **(A)** Schematic representation of the different GFP:*at*ALKBH9B fusion proteins used. **(B)** CLSM images of *N. benthamiana* leaf epidermal cells co-infiltrated with agrobacterium expressing constructs indicated in **(A)** plus the SGS3 protein fused to the mCherry fluorescent protein (mCherry:SGS3). Scale bar = 20 μm.

To identify the region of the protein involved in this subcellular localization, we performed localization experiments in *N. benthamiana* leaves by expressing a series of deletion mutants of *at*ALKBH9B fused to the GFP (Figure 6A).

Thus GFP:*at*ALKBH9B mutants were transiently co-expressed by agroinfiltration with mCherry:SGS3. As expected, full length *at*ALKBH9B co-localized with SGS3 (Figure 6B, upper panels) whereas that only *at*ALKBH9B with a deletion of the C-terminal 20 residues (Figure 6B panel GFP:*at*ALKBH9B_Δ__20Ct_) accumulated in cytoplasmic granules colocalizing with SGS3. In contrast, deletion of the N-terminal 20, C-terminal 40 or internal 186 residues (Figure 6A; GFP:*at*ALKBH9B_Δ__20Nt_, GFP:*at*ALKBH9B_Δ__40Ct_ and GFP:*at*ALKBH9B_Δ__186Int_) resulted in proteins showing a diffuse pattern throughout the cytoplasm (Figure 6B). These results indicated that different regions of the *at*ALKBH9B sequence are involved in its subcellular localization, which could be explained assuming that the proper three-dimensional folding of the protein might be a critical requirement to form the cytoplasmic granules and consequently to colocalize with siRNA bodies.

## Discussion

m^6^A RNA methylation in plants has emerged as an important cellular process of gene regulation in development (Wan et al., 2015; Růžička et al., 2017), response to abiotic stress (Li et al., 2014), and antiviral defense (Martínez-Pérez et al., 2017). Recently, *at*ALKBH9B, *at*ALKBH10B, and *at*ALKBH6 were described as m^6^A *erasers* involved in AMV infection, flowering time in *Arabidopsis*, and growth and abiotic stress responses, respectively (Duan et al., 2017; Martínez-Pérez et al., 2017; Huong et al., 2020).

To dissect the functional activity of *at*ALKBH9B in plant-virus infection, a protein-mapping analysis was carried out to identify putative domains required to regulate this process. In this context, by mutagenesis and northwestern analysis, we analyzed the ALKBH9B RNA binding activity and delimited the region between residues 427 and 467 as critical for binding *in vitro* sgRNA4 (AMV). Moreover, EMSA analysis led us to determine that the *K*_*d*_ of the binding of the RBD alone (0.64 μM, Figure 3) was slightly higher than the one obtained with the full-length protein (0.30 μM, Figure 1), evidencing that other domains of the protein favor the binding to the viral RNA. Nonetheless, the RBD *K*_d_ value obtained is within the range reported for other plant viruses RBPs, such as Nla from Tobacco etch virus -TVE- (1.1–1.3 μM) (Daròs and Carrington, 1997), Turnip crinkle virus CP (0.5 μM) (Skuzeski and Morris, 1995), p7 MP of Carnation mottle virus (0.7 μM) (Marcos et al., 1999), AMV CP (0.5 μM) (Baer et al., 1994), or the MP of prunus necrotic ringspot virus (Herranz and Pallás, 2004). Additionally, Huong et al. (2020) showed the RNA-binding capabilities of *at*ALKBH6, although their biochemical parameters were not determined.

Visual inspection and computational analysis of the *at*ALKBH9B sequence revealed no obvious structured RBD that could justify the RNA-binding properties described above. However, recently Varadi et al. (2015) demonstrated the prevalence of IDRs in RNA binding proteins and domains. Among other functions, IDRs are implicated in protein–protein and RNA–protein interactions (Castello et al., 2012). In fact, IDRs are a type of domain that is frequently found in proteins that undergo liquid–liquid phase separation (LLPS), a process that likely contributes to the formation and stability of RNA granules (Alberti et al., 2019). This has been demonstrated in YTHDF1, YTHDF2, and YTHDF3, m^6^A-binding proteins, which undergo LLPS in the presence of polymethylated mRNAs. The resulting mRNA-YTHDF complexes form P-bodies and stress granules (Ries et al., 2019). Additionally, IDRs have been found to encompass diverse functional motives, e.g., well-established RNA binding activity such as RGG/RG and YGG motives, or low-complexity (LC) domains (Castello et al., 2012; Alberti et al., 2019). Both the conformational flexibility and the establishment of extended conserved electrostatic interfaces with RNAs have been proposed to provide the capability of the IDRs to specifically target different RNAs (Varadi et al., 2015). Furthermore, many proteins localized in RNA granules contain IDRs encompassing prion-like LC domains required for RNA granules assembly (Gilks et al., 2004; Reijns et al., 2008). The *at*ALKBH9B RBD identified here is enriched with G and R (20% and 15%, respectively) and presents an RGxxxRGG motif between positions 469 and 465 (Figure 1A) or a Tri-RG motif present in proteins implicated in various cellular processes like RNA biogenesis, DNA damage signaling, and mRNA translation (Thandapani et al., 2013). Interestingly, we found that 45.4% of *at*ALKBH9B amino acid sequence forms IDRs, located at the N-terminal part, delimiting the internal AlkB-*like* domain located between positions 216 and 411 and at the C-terminal part. In fact, around 77.5% of the RBD is contained in the C-terminal disordered region (Figure 4). Furthermore, the C-terminal IDR of *at*ALKBH9B exhibits two additional RG residues at positions 473–474 and 466–497, respectively (Figure 4).

Our results can be explained considering that the RGxxxRGG motif between positions 459 and 466 plays a critical role in the protein–RNA interface. This interaction might induce the formation of a flexible structure permitting additional contacts through RG residues at positions 473–474 and 496–497 and YG residues at 506–507, enhancing the binding affinity and specificity of the interaction. For example, the splicing factor Tra2-β1 presents IDRs in the N- and C-terminal regions of the RRM. In the interface protein–RNA, this region adopts a folded structure, forming extensive contacts (Cléry et al., 2011). On the other hand, we previously reported that *at*ALKBH9B is exclusively cytoplasmic, forming discrete granules which colocalize with siRNA bodies, and some are associated with P bodies (Martínez-Pérez et al., 2017). Here we show that deletion of the first N-terminal 20 residues or the C-terminal last 40 amino acids impedes its accumulation in siRNA bodies rendering a diffuse cytoplasmic pattern (Figure 6B). Interestingly, the deleted *at*ALKBH9B N- and C-terminal parts are predicted to form IDRs, and the C-terminal is rich in Y and S residues, which would participate in the RNA granules formation. In this sense, mutation of LC domains in hnRNPA2 and FUS reduced the efficiency of their recruitment in hydrogel polymers *in vitro* (Xiang et al., 2015) and stress granules (SG) in cells (Kato et al., 2012), respectively. Moreover, phosphorylation of the SG-nucleating protein G3BP within its IDR (Ser 149) impaired its ability to induce the formation of SGs (Kedersha et al., 2016).

Finally, we found that amino acids located between positions 387 and 427 of *at*ALKBH9B are critical for CP–AMV interaction (Figure 5 and Supplementary Figure 3). Interestingly, this region is located next to the RNA-binding site, but it is not part of the C-terminal IDR (Figure 4). Considering that *at*ALKBH9B binds RNA to remove m^6^A-modification (Martínez-Pérez et al., 2017), and the CP is a multifunctional protein indispensable for the viral replication and translation (Bol, 2005; Guogas et al., 2005; Herranz et al., 2012), it may be possible that the CP binds *at*ALKBH9B in order to modulate the vRNA binding and the m^6^A demethylase activity in benefit of the virus.

**FIGURE 7:**
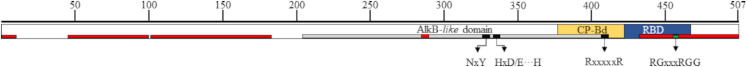
*at*ALKBH9B multi-domain structure. Predicted IDRs, between positions 1–12, 48–101, 104–187, 280–288, and 437–507, are shown in red. AlkB-*like* domain, located at 216–411, is shown in gray. Black boxes in the AlkB-*like* domain delimit the 2-OG stabilizing motif (NxY: 324–326), motif involved in binding to iron -Fe II- (HxD/E⋅⋅⋅H: 335–340) and the substrate specificity motif (RxxxxxR: 335–411) [Alignment between the *at*ALKBH9B and *hs*ALKBH5 proteins, using the BLAST tool]. The AMV CP binding domain (CP-bd: 387–427) and RNA binding domain (RBD: 427–467) are shown in yellow and blue. RGxxxRGG motif between positions 469 and 465 within the RBD characterized here is shown in green.

In summary, we have mapped the *at*ALKBH9B regions responsible for RNA and viral CP binding and those required for its localization in stress granules. CP-binding and RNA binding are located at the protein C-terminal, the former partly overlapping the AlkB-*like* domain, whereas the RBD is partially embedded in the predicted IDR located at the C-terminal (Figure 7). Thus, as found in other proteins (Protter and Parker, 2016), *at*ALKBH9B IDRs and the RBD could act cooperatively to promote the formation of RNA granules. This follows the role of both IDRs and folded domains in mediating RNA binding and oligomerization by acting together with RNAs to produce and maintain these granules (Jonas and Izaurralde, 2013; Protter and Parker, 2016). Therefore, although our results reinforce the existence of this cooperativity in RNA granules formation, the mechanisms underlying IDR-folded domain cooperativity and their potential regulation need further examination.

## Materials and Methods

### Protein Expression and Purification in Bacteria

Full-length *at*ALKBH9B ORF and deletion mutants were subcloned into pGEX-KG (GE Healthcare Life Sciences) to generate a construct with 9B merged to the C-terminal part of the GST. GST and GST:*at*ALKBH9B fusion proteins were expressed in BL21 (DE3) *E. coli* cells and purified with glutathione Sepharose 4B beads (GE Healthcare Life Sciences) according to the manufacturer’s recommendations. All protein purification procedures were performed at 4°C.

### Nucleic Acid-Binding Assay by Northwestern Blot and EMSA

Dilutions of GST or GST:*at*ALKBH9B purified proteins were electrophoresed in 12% SDS/PAGE and transferred to nitrocellulose membranes. Membranes were incubated overnight at 4°C in Renaturing Buffer (10 mM Tris⋅HCl pH 7.5, 1 mM EDTA, 0.1 M NaCl, 0.05% Triton X-100, 1X Blocking Reagent, Roche). After this, membranes were incubated with 20 mL of the same buffer containing 50 ng/μL of the AMV sgRNA 4 labeled with digoxigenin for 3 h at 25°C. For the EMSA assay, 5 ng of 3′UTR of AMV RNA 3 transcripts were heated for 5 min at 85°C and cooled at room temperature for 15 min. Different amounts of purified GST:*at*ALKBH9B fusion proteins were added and incubated for 30 min at 4°C in a 10-μl final volume of Union Buffer (100 mM Tris-HCl pH 8.0, 1 M NaCl, 8 units of RiboLock RNase inhibitor, Thermo Fisher Scientific). Following incubations, the samples were separated through 1.2% agarose. RNAs were transferred to positively charged nylon membranes (Roche). RNAs were visualized on blots using DIG-labeled riboprobes corresponding to the 3’UTR of AMV RNA 3. Synthesis of the digoxigenin-labeled riboprobes, hybridization and digoxigenin-detection procedures were carried out as described in (Pallás et al., 1998).

### Bimolecular Fluorescence Complementation and Subcellular Localization Study

^*N–C*^YFP:*at*ALKBH9B (wild-type and deletion mutants); GFP:*at*ALKBH9B and mCherry:SGS3 fusion proteins were cloned using the Gateway System (Invitrogen) according to the manufacturer’s recommendations. Plasmid expressing the CP_AMV_ merged to the N, or C-terminal part of the YFP (^*N–C*^YFP:CP_AMV_), was previously described in Aparicio et al. (2006). All binary vectors were transformed into *Agrobacterium tumefaciens* C58 cells. Pairs of cultures carrying specific fusion proteins were mixed at an optical density of 0.5 each in infiltration solution (10 mM MES, pH 5.5, and 10 mM MgCl_2_) and co-infiltrated into *N. benthamiana* leaves. Laser-scanning confocal images were taken 48 h after agroinfiltration. Excitation and emission wavelengths were 488 and 508 nm for GFP, 514 and 527 nm for YFP, and 545 and 572 nm for mCherry. Expression of fusion proteins was corroborated by western blot analysis using anti-^*Nt*^GFP or anti-^*Ct*^GFP (Roche) antibodies were conducted following the recommendations of the manufacturer.

## Data Availability Statement

The original contributions presented in the study are included in the article/Supplementary Material, further inquiries can be directed to the corresponding author.

## Author Contributions

VP and FA conceived the project and designed the experiments. LA-M conducted the experiments with assistance from JM-M and MM-P. LA-M, FA, and VP wrote the manuscript. All authors analyzed and discussed the results.

## Conflict of Interest

The authors declare that the research was conducted in the absence of any commercial or financial relationships that could be construed as a potential conflict of interest.

## Footnotes

1 http://prdos.hgc.jp/cgi-bin/top.cgi
