# Supplementary figures and images for: Mapping of Functional Subdomains in the atALKBH9B m6A-Demethylase Required for Its Binding to the Viral RNA and to the Coat Protein of Alfalfa Mosaic Virus
Source: Front Plant Sci. 2021 Jul 5;12:701683. doi: 10.3389/fpls.2021.701683 (PMC8287571; doi:10.3389/fpls.2021.701683)

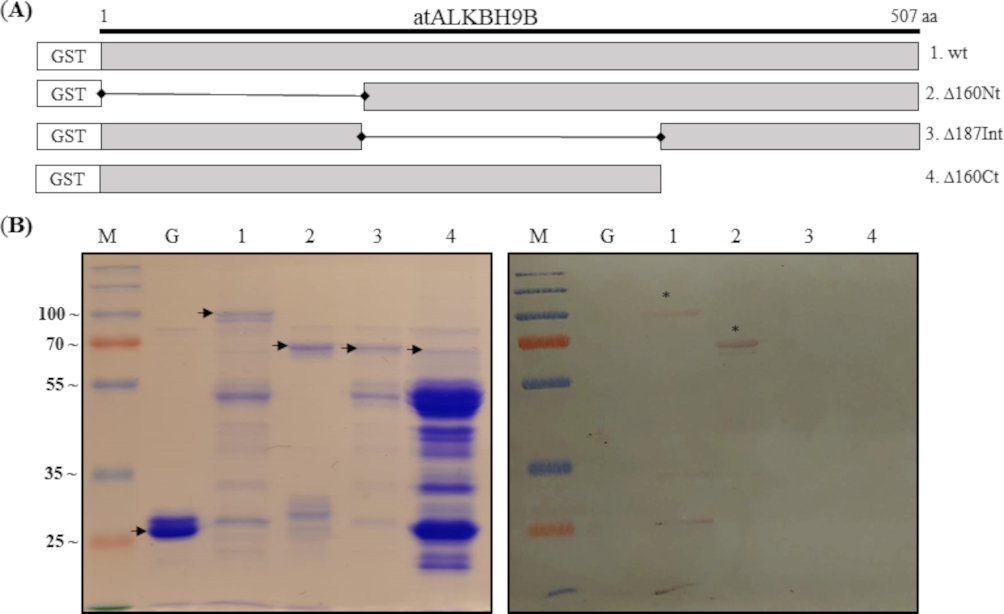

Supplement: Supplementary file 1 [file Image_1.TIF]

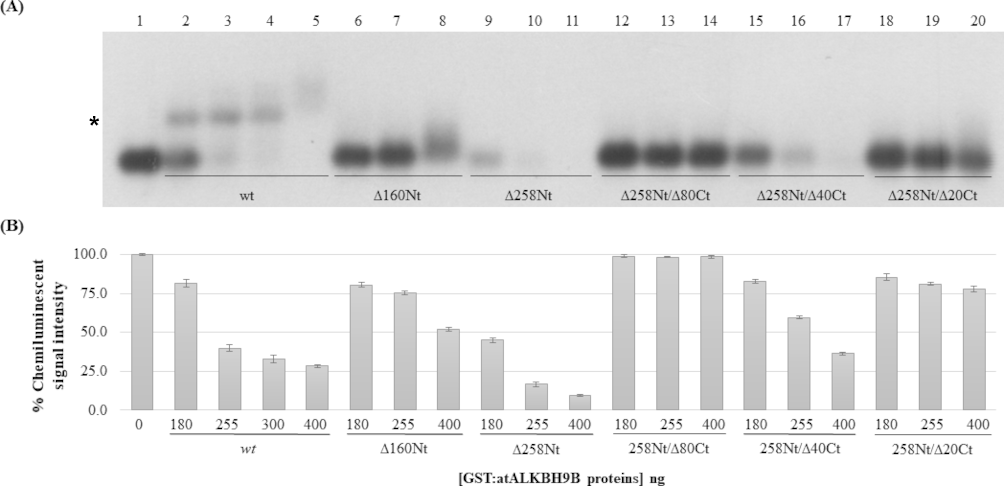

Supplement: Supplementary file 2 [file Image_2.TIF]

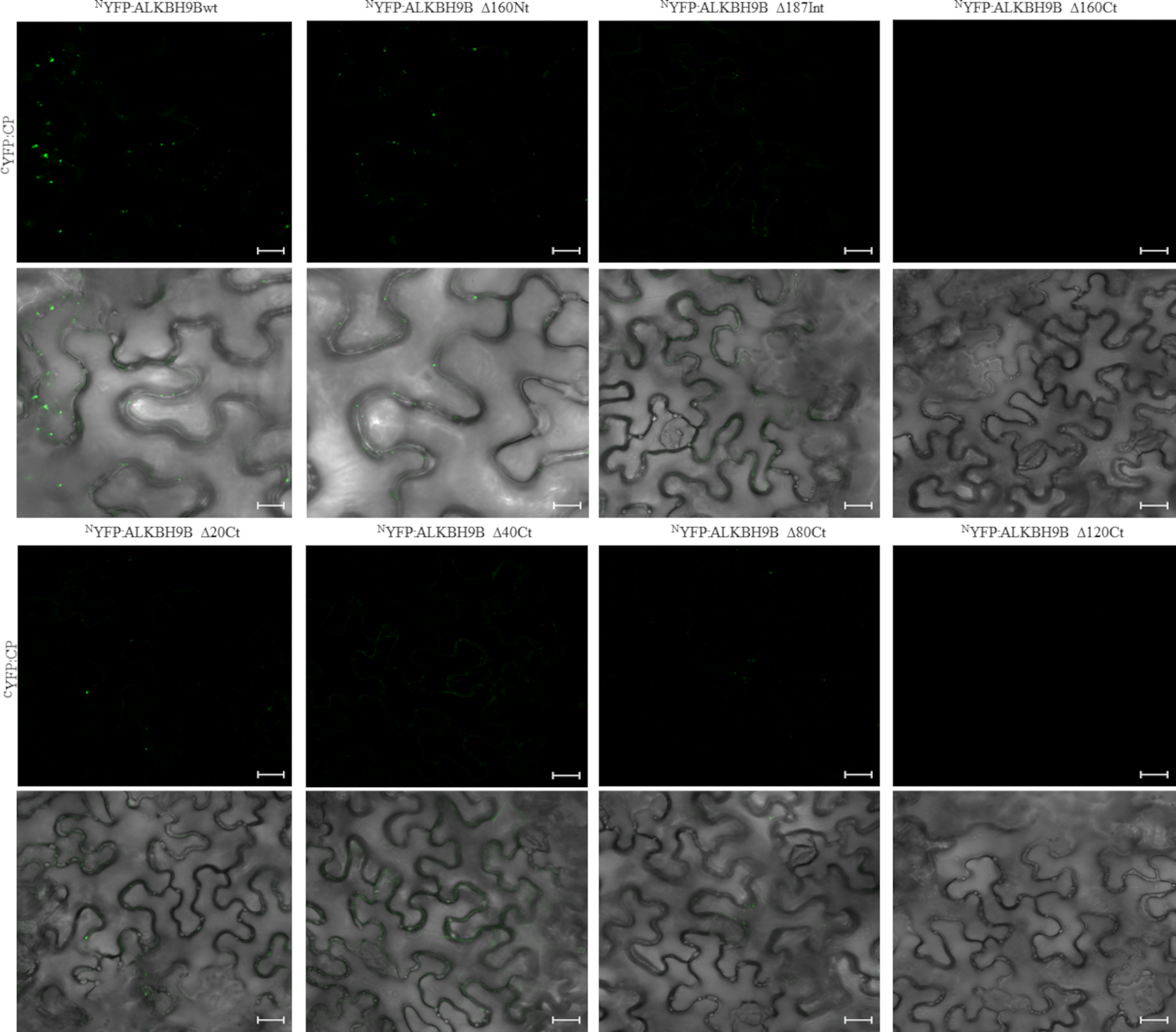

Supplement: Supplementary file 3 [file Image_3.TIF]

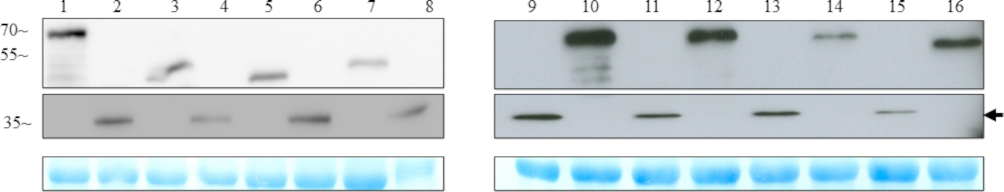

Supplement: Supplementary file 4 [file Image_4.TIF]
